# NSeq: a multithreaded Java application for finding positioned nucleosomes from sequencing data

**DOI:** 10.3389/fgene.2012.00320

**Published:** 2013-01-11

**Authors:** Abhinav Nellore, Konstantin Bobkov, Elizabeth Howe, Aleksandr Pankov, Aaron Diaz, Jun S. Song

**Affiliations:** ^1^Institute for Human Genetics, University of CaliforniaSan Francisco CA, USA; ^2^The Eli and Edythe Broad Center of Regeneration Medicine and Stem Cell Research, University of CaliforniaSan Francisco CA, USA; ^3^Department of Epidemiology and Biostatistics, University of CaliforniaSan Francisco CA, USA; ^4^Department of Bioengineering and Therapeutic Sciences, University of CaliforniaSan Francisco CA, USA

**Keywords:** nucleosome, nucleosome positioning

## Abstract

We introduce NSeq, a fast and efficient Java application for finding positioned nucleosomes from the high-throughput sequencing of MNase-digested mononucleosomal DNA. NSeq includes a user-friendly graphical interface, computes false discovery rates (FDRs) for candidate nucleosomes from Monte Carlo simulations, plots nucleosome coverage and centers, and exploits the availability of multiple processor cores by parallelizing its computations. Java binaries and source code are freely available at https://github.com/songlab/NSeq. The software is supported on all major platforms equipped with Java Runtime Environment 6 or later.

## 1. Introduction

Eukaryotic DNA is organized into chromatin, consisting of repeating nucleosomes adjoined by linker DNA. A nucleosome itself is composed of ~146 bp of DNA wound around an octameric histone core. The core histones participate in the epigenetic regulation of gene expression in two important ways: they can block access to regulatory sequences by DNA-binding factors; and covalent modifications of their N-terminal tails can affect the recruitment of protein complexes, which in turn influences transcriptional regulation. Although nucleosomes are highly dynamic, being subjected to thermal fluctuations and ATP-dependent remodeling (Blossey and Schiessel, 2011), some nucleosomes are well-localized across populations of a given cell type. Such positioned nucleosomes are likely to be more functional than delocalized nucleosomes and may be under selection and regulatory forces (Yuan et al., 2005; Ozsolak et al., 2007; Field et al., 2008; Song et al., 2008).

This paper introduces NSeq, an open-source Java application that rigorously identifies positioned nucleosomes from the next-generation sequencing of micrococcal nuclease (MNase)-digested mononucleosomal DNA. MNase preferentially cuts linker DNA, leaving nucleosomal DNA largely intact. This ideally gives rise to clusters of reads on both sides of a positioned nucleosome, with the mean 5′-end positions of reads in the forward- and reverse-strand clusters separated by ~146 bp. NSeq uses a novel statistical test to identify positioned nucleosomes from these reads.

The organization of the rest of this paper is as follows. The next two sections highlight the distinctive features of NSeq and compare it with competing nucleosome sequencing offerings. The Usage section serves as a short user's guide to our software. The “Methods” section provides a detailed description of NSeq's algorithm.

## 2. Distinctive features

Publicly available software that also finds nucleosomes from sequencing data includes NOrMAL (Polishko et al., 2012), Template Filter (Weiner et al., 2010), PING (Zhang et al., 2012), nucleR (Flores and Orozco, 2011), and NPS (Zhang et al., 2008a). NSeq has several advantages over these alternatives:

- NSeq automatically controls for false positive positioned nucleosome calls and computes a false discovery rate (FDR). A candidate nucleosome is excluded if its FDR is above a user-specified cutoff. NOrMAL, Template Filter, and PING associate measures of confidence with nucleosomes, but their connection to FDR, if any, must be inferred by the user.
- NSeq has both user-friendly graphical and command-line interfaces. NOrMAL, NPS, and Template Filter are solely command-line utilities, while nucleR and PING run in R.
- NSeq accepts alignment data in BAM, SAM, and BED file formats. Template Filter and NOrMAL use non-standard file formats. NPS opens only BED files. PING and nucleR support input data in BAM, SAM, and BED formats through shortread, an R/Bioconductor package.
- Unlike other software discussed here, NSeq has an integrated plotting tool that displays nucleosome coverage and center positions as well as raw read positions. NSeq also outputs a WIG file with nucleosome center positions.
- NSeq is multithreaded: it exploits the availability of multicore processors, parallelizing its nucleosome search and FDR computations to improve performance. PING also supports parallel processing of input data (through the R package snowfall), but others do not.
- NSeq is fast. We ran NSeq and NOrMAL on chromosome 10 of human data (Schones et al., 2008) for default values of all parameters on an Intel i5 2.7 GHz CPU. NSeq took 133 s to process the chromosome, while NOrMAL took 9.98 days.

## 3. Software comparison

**Figure 1:**
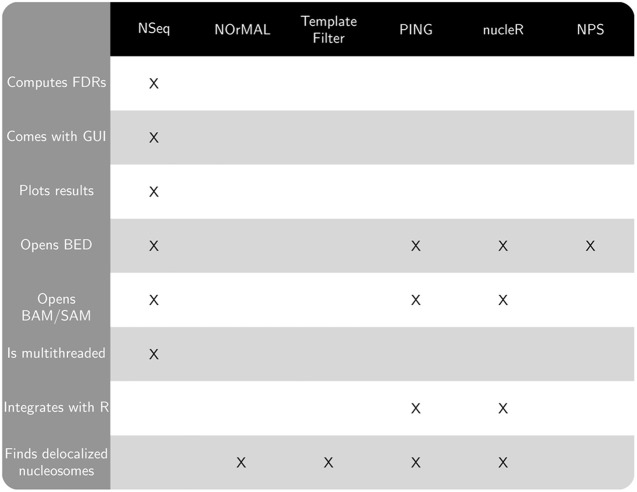
**Chart comparing features of NSeq and other publicly available nucleosome sequencing software**.

Figure 1 compares the features of the aforementioned software packages. Many of these features pertain to usability. NOrMAL was released in June 2012, and it is currently the latest publicly available nucleosome sequencing analysis software at the time of this manuscript's preparation. In its accompanying paper (Polishko et al., 2012), Template Filter is described as the current state-of-the-art in nucleosome detection software. The remainder of this section compares the accuracy of NSeq, NOrMAL, and Template Filter in finding positioned nucleosomes.

Template Filter comes with seven characteristic distributions (“templates”) of reads flanking a nucleosome. Each template is designed to match a pattern of reads on a single strand that ostensibly indicates the presence of a nucleosome. So there are 7 × 7 = 49 combinations of forward- and reverse-strand templates. The software advances a sliding window across a genome to analyze read count data, computing cross-correlations with the 49 combinations of forward- and reverse-strand templates for various spacings between template pairs. This yields a correlation heat map for each template pair, with template pair spacing and window position on the axes. Local maxima are associated with candidate nucleosomes. A greedy algorithm then selects the best assignment of nucleosomes.

The seven templates that come with Template Filter were obtained by applying the procedure outlined in the previous paragraph to a *Saccharomyces cerevisiae* dataset, but using a single Gaussian-shaped template. That is, a single cross-correlation was calculated for a given window using Gaussian-shaped ansatzes to characterize the forward- and reverse-strand read accumulations. The read patterns of these nucleosomes were then clustered using *k*-means clustering. Each of the seven templates was chosen from a different cluster of read patterns.

NOrMAL uses a mixture model of *k* Gaussians per chromosome to probabilistically model nucleosome occupancy and applies an expectation-maximization (EM) algorithm to learn the parameters from read count data. Each Gaussian corresponds to a candidate nucleosome. NOrMAL's output associates confidence and fuzziness scores with each nucleosome. The fuzziness scores are parameters from the mixture model. The lower the fuzziness score, the better-positioned a nucleosome; the lower the confidence score, the more likely a nucleosome is a false discovery.

The number *k* of Gaussian clusters in NOrMAL's mixture model is found by following these steps:

1. *k* is set equal to the size of a chromosome divided by the expected size of a nucleosome, an underestimated parameter specified by the user.
2. The EM algorithm mentioned above is run until it converges.
3. Distances between Gaussian clusters are checked, and clusters are merged if they overlap above a threshold input by the user.

Steps 2 and 3 are repeated until clusters are no longer merged.

We ran Template Filter, NOrMAL, and NSeq on nucleosome sequencing data for *S. cerevisiae* (Tsankov et al., 2010). Default values of all parameters were used. NSeq found 28,896 positioned nucleosomes in the data. NOrMAL found 49,218 nucleosomes, and the distribution of their fuzziness scores peaked at 15; we thus considered as positioned those nucleosomes whose fuzziness scores were less than 15. We then simulated delocalized nucleosomes as follows: for each read, a random integer was drawn from the uniform distribution on {−73, …, 73} and added to its position. Since a sharply positioned nucleosome is associated with tightly clustered reads, shaking the original data in this random manner removes the characteristic signatures of localized nucleosomes; a good algorithm for detecting positioned nucleosomes should not call many candidates in such simulated data. We stress that our simulations do not merely add noise to the original data; they effectively reconstruct the data so the signal-to-noise ratio is nearly zero. While a good nucleosome detector should be robust to noise, it should not often mistake noise for signal.

**Figure 2:**
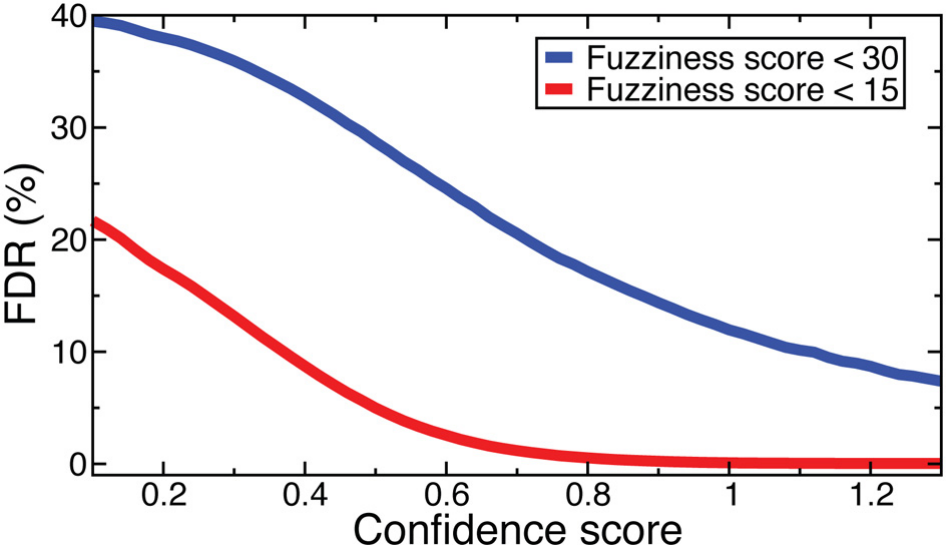
**FDRs computed from NOrMALÕs results using simulations described in text at fuzziness score thresholds 30 (blue) and 15 (red)**.

Denote as *C*(*f*, *s*) the criteria that fuzziness is less than *f* and confidence score is greater than *s*. FDRs were computed for NOrMAL as the average ratio of the total number of nucleosomes satisfying *C* in simulated data to that in the original data. The FDRs corresponding to *C*(15, *s*) and *C*(30, *s*) are displayed in Figure 2. Note that the FDR decreases to close to zero for fuzziness scores <15 as the confidence score increases, indicating that low fuzziness score and high confidence score are generically associated with true positive positioned nucleosomes. An FDR was computed for NSeq just as for NOrMAL, but without the criteria *C*, giving 1.31 × 10^−3^%.

Template Filter found 64,990 nucleosomes in the original *S. cerevisiae* data, and the distribution of their correlation coefficients peaked at 0.9. Denote as *D*(*F*, *R*) the criteria that a nucleosome has correlation coefficient >0.9, and is associated with template *F* on the forward-strand and template *R* on the reverse-strand, with *F*, *R* ∈ {1,…, 7}. We computed FDRs for Template Filter as for NOrMAL but with the criteria *D* rather than *C*. The median FDR across the 49 forward-reverse template combinations was 36.5%, with a median absolute deviation of 8.50%. The minimum FDR of 25.3% occurred for *D*(4, 4).

Template Filter thus lacks the efficacy of both NSeq and NOrMAL for reliably identifying positioned nucleosomes, and NOrMAL requires the user to manually estimate the FDR.

## 4. Usage

NSeq is distributed as a Java Archive (NSeq.jar) and can be run on any machine equipped with Java Runtime Environment 6 or later. Netbeans IDE 7.0.1 was used to design the graphical user interface (GUI), which consists of standard Swing components. To start the GUI and set a maximum Java Virtual Machine heap size of 2 GB, enter

java -jar -Xmx2g NSeq.jar

at any Windows or Unix-like command prompt. For processing large genomes like human and mouse, NSeq should be run with a maximum heap size of at least 10 GB:

java -jar -Xmx10g NSeq.jar.

### 4.1. Startup screens

**Figure 3:**
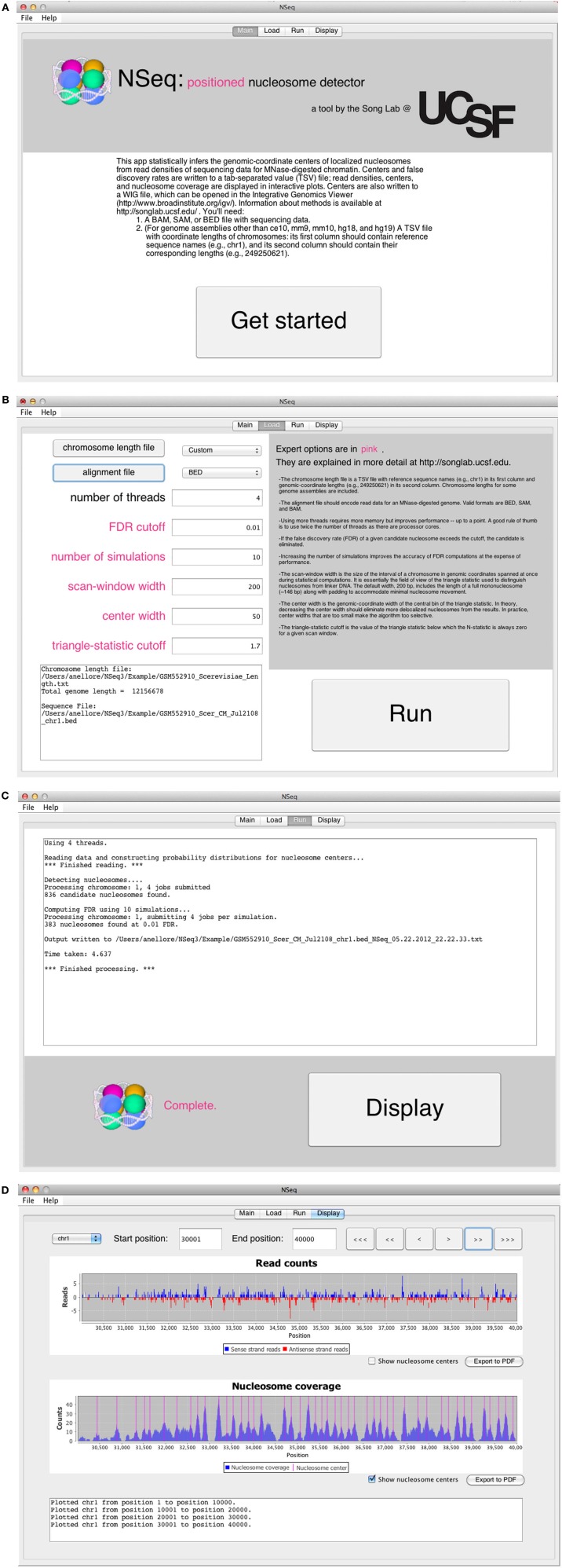
**(A)** NSeq’s welcome screen. **(B)** NSeq’s load screen. **(C)** NSeq’s run screen. **(D)** NSeq’s display screen.

The opening screen (Figure 3A) explains the purpose of the application and the inputs required of the user. NSeq analyzes alignment data in BAM, SAM, or BED format. It assumes that the data are single-end. To facilitate fast reading of aligned data, a tab-separated value (TSV) file with chromosome lengths is required for genome assemblies other than ce10, mm9, mm10, hg18, and hg19. This is a text file with reference sequence names (e.g., chr1) in its first column and chromosome lengths (e.g., 249,250,621) in its second column; it is used to preallocate memory for storing read data. Click “Get started” to proceed to the load screen (Figure 3B). Here, the user specifies the locations of the chromosome-length and alignment files, as well as parameters used by NSeq in its nucleosome search. The number *T* of threads controls the extent to which the computation is parallelized. When *T* = 1, the computation is not parallel. We recommend using a value of *T* at least as large as the number of available CPU cores; we found that computations are fastest when *T* is about twice the number of available cores. The default FDR cutoff *F* is 0.01. If the FDR computed for a given candidate nucleosome is above *F*, the nucleosome is excluded from all results. Other parameters that can be toggled on the load screen are explained in the gray help box as well as in “Methods.” Most users will find the default settings adequate.

### 4.2. Analyzing alignment data

NSeq starts searching for nucleosomes using the alignment data after “Run” is clicked on the load screen. Status updates are displayed in a text box (Figure 3C). Each chromosome is divided into overlapping intervals; chromosomes are analyzed interval by interval, and different intervals are assigned to different threads. When NSeq has finished its analysis, two files are written in the same directory as the alignment data. One is a text (TXT) file with genomic coordinates and FDR estimates of nucleosome centers, which correspond to the centers of scan windows with non-zero *N*-statistics. (Our algorithm is described in detail in “Methods.”) The second file written is a WIG file that can be opened in the Integrated Genome Browser (Nicol et al., 2009). It contains nucleosome centers and their associated triangle statistics. The parts of the filenames that precede their extensions have the format [alignment filename]_[datestamp]_[timestamp].

### 4.3. Displaying results

Clicking “Display” on the run screen brings up a histogram of raw read positions and an overlay of nucleosome center positions on a plot of nucleosome coverage (Figure 3D). A genome can be navigated by changing the chromosome number as well as the start and end coordinates of the plots. In the read-position histogram, positive-strand read counts are in blue, and negative-strand read counts are in red. The nucleosome coverage plot is obtained as described in Zhang et al. (2008b): NSeq extends a positive-strand read by 75 bases to the right, and a negative-strand read by 75 bases to the left. Here, “extend” means that each of the counts at included coordinates is incremented by 1. The extended counts are then shifted by 37 bases to the right for positive-strand reads and 37 bases to the left for negative-strand reads. This gives rise to accumulations of counts near nucleosome centers, which are denoted by red lines in the bottom panel in Figure 3D.

### 4.4. Command-line interface

NSeq can also be used at the command-line just to obtain the output TXT and WIG files. This option may be preferred for batching jobs on a cluster. For information on the command-line interface, enter

java -jar NSeq.jar -h

at a prompt.

## 5. Methods

**Figure 4:**
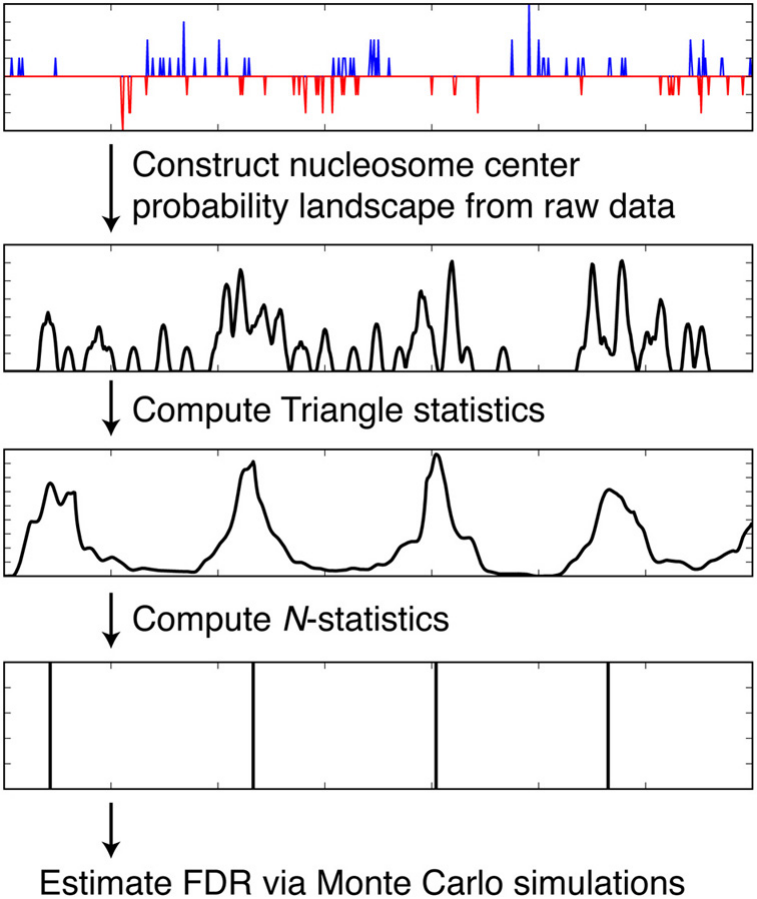
**Steps taken by NSeq during nucleosome detection.** NSeq runs through the steps summarized here to extract nucleosome centers from raw sequencing data. The plots in black representatively depict quantities computed across the genome.

We discuss how NSeq finds nucleosome centers in this section. The steps are summarized in Figure 4. Parameters from the load screen (Figure 3B) explained here are the scan-window width *W*, the center width *B*_*w*_, the critical triangle-statistic cutoff *t*_*c*_, the FDR cutoff *F*, and the number of simulations *S*.

### 5.1. Converting reads into a nucleosome center probability landscape

The alignment data are a snapshot of nucleosome locations in random samples from a cell population. But no nucleosome is exactly static with respect to DNA, and even well-localized nucleosomes experience small shifts. In addition, a given read should correspond to one end of a nucleosome in one cell, but there is inherently some uncertainty in where linker DNA is cleaved by MNase. [Indeed, MNase cleavage sites are known to have a bias toward AT-rich regions (Horz and Altenburger, 1981)]. Consider a histogram of read counts whose bins are genomic-coordinate positions. A positive-strand alignment starts at the 5′ end of the reference sequence and extends to the right; a negative-strand alignment starts at the 3' end of the reference sequence and extends to the left. So there should be minimally spread accumulations of reads (more precisely, alignment start-position counts) on either side of a positioned nucleosome.

**Figure 5:**
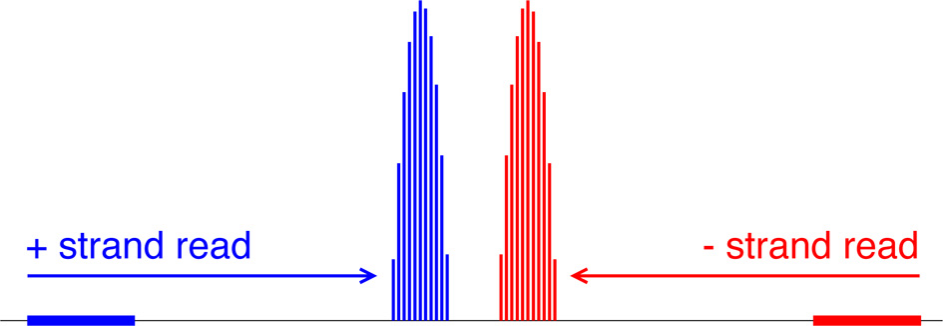
**Mapping reads to a relative probability distribution of nucleosome centers.** A positive-strand alignment is mapped to a discretized beta distribution whose leftmost bin is 68 bins to the right of the read’s start position (blue). A negative-strand alignment is mapped to a discretized beta distribution whose rightmost bin is 68 bins to the left of the read’s start position (red).

Our algorithm first converts each read location into a probability distribution of the corresponding nucleosome center and attempts to capture the uncertainty in where the linker DNA is cut (Figure 5). Suppose *y*_*i*_ is the 5′-end position of the *i*th read; then, allowing for 5 bp of ambiguity in either direction, we model the center of the corresponding nucleosome as a random variable *Z*_*i*_ = *y*_*i*_ + *X*, where *X* ∈ {68, …, 78} and *P*(*X* = *x*) is obtained from discretizing the beta distribution:

(1) $$P(X=x)=\frac{\int_{x}^{x\;+\;1}{\left(t-68\right)}^{\alpha \;-\;1}{\left(79-t\right)}^{\beta \;-\;1}dt}{B(\alpha ,\beta ){\left(11\right)}^{\alpha \;+\;\beta \;-\;1}}.$$

Our choice of the beta distribution is strategic: we require an analytic distribution with a finite domain, flexible enough to describe the empirical distribution of nucleosomal DNA lengths, yet also fast to sample from. The parameter values 68 and 78 were selected to accommodate the 5-base-pair ambiguity from the expected nucleosome center located at 146/2. For a negative-strand read at *y*_*j*_, we model its center as *Z*_*j*_ = *y*_*j*_ − *X*. NSeq estimates α and β by using previously published paired-end nucleosome sequencing data for *S. cerevisiae* (Henikoff et al., 2011). In these data, a given read pair should flank a full nucleosome. The genomic-coordinate distance between the reads in each pair was halved to obtain an empirical distribution of distances between reads and their corresponding probable nucleosome centers. We then obtained the maximum-likelihood estimates (MLEs) of α = 1.9204 and β = 1.8937, by numerically solving the equations

$$\frac{1}{N}\log\left(\prod_{i\;=\;1}^{N}\frac{t_{i}-68}{11}\right)=\psi (\alpha )-\psi (\alpha +\beta )$$

and

$$\frac{1}{N}\log\left(\prod_{i\;=\;1}^{N}\frac{79-t_{i}}{11}\right)=\psi (\beta )-\psi (\alpha +\beta ),$$

where ψ is a digamma function, and *N* is the total number of paired-end reads with their center location *t*_*i*_ in the range {68, …, 78}; we used the Scipy optimize.fsolve module with default parameters. This approach thus models the fuzziness in nucleosome-center position for every read in the alignment data. The center probability densities are then summed at each genomic location *k* and provide a score *s*_*k*_ defined as

(2) $$s_{k}=\sum_{i\;=\;1}^{R}P(Z_{i}=k),$$

where *R* is the total number of reads, resulting in a relative probability landscape for the presence of nucleosome centers across the genome.

### 5.2. Triangle statistic on the nucleosome center probability landscape

**Figure 6:**
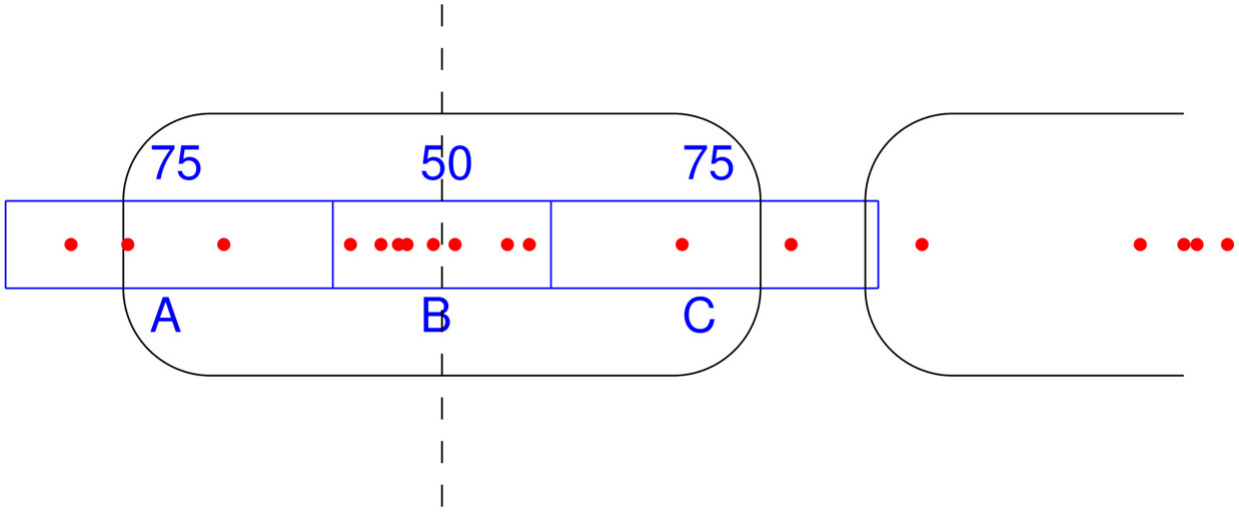
**How the triangle statistic works.** Red dots denote the centers of discretized beta distributions corresponding to reads, and rounded rectangles denote nucleosomes. The triangle statistic divides a scan-window into regions A, B, and C of sizes (in bp) 75, 50, and 75, respectively. The greater the number of dots in B (more precisely, the sum of the probability masses at positions spanned by B) compared to the number of dots in both A and C, the greater the triangle statistic.

NSeq identifies nucleosomes by advancing a scan-window that spans *W* bins across the aforementioned nucleosome-center relative probability landscape bin-by-bin, where each bin spans one basepair. Thus, two successive scan windows overlap by *W* − 1 bins. A positioned nucleosome in the landscape should appear as clustered probability masses. We assess the statistical significance of such clustering by using what we call the triangle statistic, which is motivated by the scan statistic. Suppose we partition the scan-window of length *W* = 200 into disjoint sub-windows of lengths 75, 50, and 75 (Figure 6). The triangle statistic to be defined below detects significant accumulation of nucleosome center probability mass in the central 50 bp sub-window, which allows for roughly two superhelical turn ambiguity in either direction. Although *W* = 200 by default, NSeq allows the user to change its value.

Denote as *A*, *B*, and *C* the sums of probability masses in the first, second, and third sub-windows, respectively; and, let *A*_*w*_, *B*_*w*_, and *C*_*w*_ be the corresponding lengths of sub-windows. By default, *A*_*w*_ = 75, *B*_*w*_ = 50, and *C*_*w*_ = 75. Now, consider the odds *B*/*A* and *B*/*C*. For a uniform distribution of counts across the scan-window, *B*/*A* and *B*/*C* should both approach 2*B*_*w*_/(*W* − *B*_*w*_) = 50/75. However, the presence of a positioned nucleosome preferentially puts probability masses in the central sub-window, so that both *B*/*A* and *B*/*C* are large compared to the null value of 2*B*_*w*_/(*W* − *B*_*w*_). The significance of this distortion is measured by our triangle statistic

(3) $$t=\frac{\min(B/A,B/C)}{2B_{w}/(W-B_{w})}.$$

We call *t* the triangle statistic, because it is typically much larger than 1 for peaks in the probability landscape that look, roughly, like triangles. Importantly, by construction, the triangle statistic is small when the scan-window is centered around linker DNA or delocalized nucleosomes.

### 5.3. Improving the estimates of triangle statistic

*B*/*A* and *B*/*C* are MLEs of the odds. The MLE estimates have high variance when *A*, *B*, or *C* is small; NSeq thus uses median-unbiased estimates (MUE) which are known to be more robust and accurate for small sample data (Hirji et al., 1989; Parzen et al., 2002). NSeq uses the MUE of odds ratios in the triangle statistic calculations.

Estimating the odds can be mapped to the following problem: given a Bernoulli random variable with success probability *p*, estimate *r* = *p*/(1 − *p*) from *m* successes out of total *M* i.i.d. trials. In our problem, both *m* and *M* are non-negative real numbers, but the formalism described below has a natural generalization to the continuous case. The MLE of *r* is *m*/(*M* − *m*). The MUE of *R* instead uses a MUE $\hat{p}$ of the success probability *p* to form the odds $\hat{p}/(1-\hat{p})$. The MUE $\hat{p}$ satisfies the probability conditions

$$Pr(\hat{p}\geq p)\geq \frac{1}{2}\text{ and }Pr(\hat{p}\leq p)\geq \frac{1}{2}.$$

Note that the number of successes after *M* Bernoulli trials is a sufficient statistic *m*_*s*_ and is drawn from the binomial distribution. An alternative formulation of the MUE is obtained by considering the distribution of the sufficient statistic *m*_*s*_. For the observed value *m*_*s*_ = *m*, the MUE $\hat{p}$ occurs where (Hirji et al., 1989)

(4) $$Pr(m_{s}\leq m|p=\hat{p})\geq \frac{1}{2}\text{ and }Pr(m_{s}\geq m|p=\hat{p})\geq \frac{1}{2}.$$

In general, some range of $\hat{p}$ satisfies the conditions, and the midpoint between its boundary values ${\hat{p}}_{1}$ and ${\hat{p}}_{2}$ is taken as the MUE. Each of the boundary values ${\hat{p}}_{1}$ and ${\hat{p}}_{2}$ occurs where one of the inequalities (Equation 4) is saturated:

(5) $$\begin{array}{l} Pr(m_{s}\leq m|{\hat{p}}_{1})=\sum_{m_{s}\;=\;0}^{m}\frac{M!}{(M-m_{s})!m_{s}!}{\hat{p}}_{1}^{m_{s}}{\left(1-{\hat{p}}_{1}\right)}^{M\;-\;m_{s}}=\frac{1}{2};\\ Pr(m_{s}\geq m|{\hat{p}}_{2})=\sum_{m_{s}\;=\;m}^{M}\frac{M!}{(M-m_{s})!m_{s}!}{\hat{p}}_{2}^{m_{s}}{\left(1-{\hat{p}}_{2}\right)}^{M\;-\;m_{s}}=\frac{1}{2}. \end{array}$$

The normalized incomplete beta function *I*_*x*_(α, β) is defined as

$$I_{x}(\alpha ,\beta )=\frac{\int_{0}^{x}u^{\alpha \;-\;1}{\left(1-u\right)}^{\beta -1}du}{\int_{0}^{1}u^{\alpha \;-\;1}{\left(1-u\right)}^{\beta \;-\;1}du}.$$

Equation (5) can be rewritten in terms of *I* as

$$\begin{array}{l} I_{{\hat{p}}_{1}}(m+1,M-m)=\frac{1}{2};\\ I_{{\hat{p}}_{2}}(m,M-m+1)=\frac{1}{2}. \end{array}$$

These relations are numerically solvable for ${\hat{p}}_{1}$ and ${\hat{p}}_{2}$ even for non-integer values of *M* and *m*, and the MUE $\hat{p}$ is determined from

$$\hat{p}=\frac{{\hat{p}}_{1}+{\hat{p}}_{2}}{2}.$$

To compute the triangle statistic, NSeq uses this formalism with *m* = *B* and *M* = *A* + *B*, or *m* = *B* and *M* = *C* + *B*.

### 5.4. Removing correlations among adjacent triangle statistics

Triangle statistics corresponding to scan windows that have substantial overlap are correlated. For the *S. cerevisiae* data considered in Results and other nucleosome sequencing data, we found that the autocorrelation length is 20–30 bins when *W* = 200. Several successive scan windows covering a single localized nucleosome will thus all return large values of the triangle statistic. This correlation is a problem for FDR estimation procedures, which often assume independent samples of random variables. We thus need to modify the triangle statistic so that only one of the correlated windows would yield a significant statistic for a positioned nucleosome. A similar problem arises in the analysis of ChIP-chip or ChIP-seq data, and a Poisson-clumping approach was previously used to remove the positive correlation (Zhang, 2008). Inspired by that method, we define a new statistic *N* in terms of the triangle statistics. Let *t*_*i*_ be the triangle statistic for the scan-window whose leftmost bin position is *i*. Define the new statistic *N*_*i*_ for the *i*th scan-window as

(6) $$N_{i}=\left(\prod_{j\;=\;i\;-\;25}^{i\;-\;1}I(t_{j}<t_{i})\right)\left(\prod_{j\;=\;i\;+\;1}^{i\;+\;26}I(t_{i}\geq t_{j})\right)I(t_{i}\geq t_{c})\times I\left(\sum_{j\;=\;1}^{10}t_{i\;+\;68\;+\;j}\leq 10\right)I\left(\sum_{j\;=\;1}^{10}t_{i\;-\;78\;+\;j}\leq 10\right)\text{​},$$

where *I* denotes an indicator function, and *t*_*c*_ is a critical cutoff. *N*_*i*_ is either 1 or 0, and the *N*_*i*_ for successive windows are *anticorrelated*. Moreover, at most only one *N*_*j*_ ∈ {*N*_*i* − 25_, …, *N*_*i* + 25_} is non-zero; the *N*-statistic picks out clumps of scan windows with large triangle statistics. The last two indicator functions set *N*_*i*_ = 0 when there are similar clumps in the neighborhood of the *i*th window; they provide extra insurance against the possibility of detecting overlapping or delocalized nucleosomes. In NSeq, the critical cutoff *t*_*c*_ is by default 1.7, which we found to be sufficiently low to detect all nucleosomes below FDR 0.01. NSeq nominates all scan windows for which *N*_*i*_ = 1 as candidate positioned nucleosomes, and then filters out candidates which are above the specified FDR cutoff *F*, as described below. Both *F* and *t*_*c*_ can be toggled in the load window.

### 5.5. Computing false discovery rates

An FDR associated with a candidate nucleosome is found by performing the following steps:

**Table:** 

FDR Estimation
Let *S* = number of simulations;
Let *R* = total number of reads in the sequencing data;
Let *t*_*j*_ = triangle statistic associated with the *j*th candidate nucleosome.
Let *M*_*j*_ = number of candidate nucleosomes with triangle statistic ≥ *t*_*j*_.
For *k* = 1,…, S:
For *i* = 1, …, *R*:
Sample *X*~ uniform distribution on {−73,…, 73}.
Shift *i*th read by *X* to simulate delocalized nucleosomes.
Run NSeq on the simulated data.
Set *m*_*kj*_ = number of nucleosomes with triangle statistic ≥ *t*_*j*_ in the simulated data.
Set FDR(*t*_*j*_) = ∑^*nsim*^_*k*= 1_ *m*_*kj*_/*M*_*j*_.

### Conflict of interest statement

The authors declare that the research was conducted in the absence of any commercial or financial relationships that could be construed as a potential conflict of interest.

## References

[B1] Blossey R. Schiessel H. (2011). The dynamics of the nucleosome: thermal effects, external forces and ATP. FEBS J. 278, 3619–3632 10.1111/j.1742-4658.2011.08283.x 21812931

[B2] Field Y. Kaplan N. Fondufe-Mittendorf Y. Moore I. Sharon E. Lubling Y. (2008). Distinct modes of regulation by chromatin encoded through nucleosome positioning signals. PLoS Comput. Biol. 4:e1000216 10.1371/journal.pcbi.1000216 18989395 PMC2570626

[B3] Flores O. Orozco M. (2011). nucleR: a package for non-parametric nucleosome positioning. Bioinformatics 27, 2149–2150 10.1093/bioinformatics/btr345 21653521

[B4] Henikoff J. Belsky J. Krassovsky K. MacAlpine D. Henikoff S. (2011). Epigenome characterization at single base-pair resolution. Proc. Natl. Acad. Sci. U.S.A. 108, 18318–18323 10.1073/pnas.1110731108 22025700 PMC3215028

[B5] Hirji K. Tsiatis A. Mehta C. (1989). Median unbiased estimation for binary data. Am. Stat. 43, 7–11

[B6] Horz W. Altenburger W. (1981). Sequence specific cleavage of DNA by micrococcal nuclease. Nucleic Acids Res. 9, 2643–2658 10.1093/nar/9.12.2643 7279658 PMC326882

[B7] Nicol J. Helt G. Blanchard S. Raja A. Loraine A. (2009). The Integrated Genome Browser: free software for distribution and exploration of genome-scale datasets. Bioinformatics 25, 2730–2731 10.1093/bioinformatics/btp472 19654113 PMC2759552

[B8] Ozsolak F. Song J. Liu X. Fisher D. (2007). High-throughput mapping of the chromatin structure of human promoters. Nat. Biotechnol. 25, 244–248 10.1038/nbt1279 17220878

[B9] Parzen M. Lipsitz S. Ibrahim J. Klar N. (2002). An estimate of the odds ratio that always exists. J. Comput. Graph. Stat. 1, 420–436

[B10] Polishko A. Ponts N. Le Roch K. Lonardi S. (2012). NORMAL: accurate nucleosome positioning using a modified Gaussian mixture model. Bioinformatics 28, i242–i249 10.1093/bioinformatics/bts206 22689767 PMC3371838

[B11] Schones D. Cui K. Cuddapah S. Roh T. Barski A. Wang Z. (2008). Dynamic regulation of nucleosome positioning in the human genome. Cell 132, 887–898 10.1016/j.cell.2008.02.022 18329373 PMC10894452

[B12] Song J. Liu X. Liu X. He X. (2008). A high-resolution map of nucleosome positioning on a fission yeast centromere. Genome Res. 18, 1064–1072 10.1101/gr.075374.107 18411404 PMC2493395

[B13] Tsankov A. Thompson D. Socha A. Regev A. Rando O. (2010). The role of nucleosome positioning in the evolution of gene regulation. PLoS Biol. 8:e1000414 10.1371/journal.pbio.1000414 20625544 PMC2897762

[B14] Weiner A. Hughes A. Yassour M. Rando O. Friedman N. (2010). High-resolution nucleosome mapping reveals transcription-dependent promoter packaging. Genome Res. 20, 90–100 10.1101/gr.098509.109 19846608 PMC2798834

[B15] Yuan G. Liu Y. Dion M. Slack M. Wu L. Altschuler S. (2005). Genome-scale identification of nucleosome positions in *S.* cerevisiae. Science 309, 626–630 10.1126/science.1112178 15961632

[B16] Zhang X. Robertson G. Woo S. Hoffman B. Gottardo R. (2012). Probabilistic inference for nucleosome positioning with MNase-based or sonicated short-read data. PLoS ONE 7:e32095. 10.1371/journal.pone.0032095 22393380 PMC3290535

[B17] Zhang Y. (2008). Poisson approximation for significance in genome-wide ChIP-chip tiling arrays. Bioinformatics 24, 2825–2831 10.1093/bioinformatics/btn549 18953047

[B18] Zhang Y. Shin H. Song J. Lei Y. Liu X. (2008a). Identifying positioned nucleosomes with epigenetic marks in human from ChIP-Seq. BMC Genomics 9:537 10.1186/1471-2164-9-537 19014516 PMC2596141

[B19] Zhang Y. Shin H. Song J. Lei Y. Liu X. (2008b). Nucleosome positioning from sequencing. http://liulab.dfci.harvard.edu/NPS/

